# Linking Hippocampal Atrophy to Emotional Dysregulation and Sleep Disturbances in Neurodegenerative Disease

**DOI:** 10.1002/alz70857_100342

**Published:** 2025-12-24

**Authors:** Kaela A. Franco, Joel Ramirez, Fuqiang Gao, Vanessa Yhap, Christopher JM Scott, Maged Goubran, Mark I. Boulos, Sandra E. Black, ONDRI Investigators

**Affiliations:** ^1^ Dr. Sandra Black Centre for Brain Resilience and Recovery, Sunnybrook Research Institute, Toronto, ON, Canada; ^2^ Dr. Sandra E. Black Centre for Brain Resilience and Recovery, LC Campbell Cognitive Neurology, Hurvitz Brain Sciences Program, Sunnybrook Research Institute, University of Toronto, Toronto, ON, Canada; ^3^ University of Toronto, Toronto, ON, Canada; ^4^ Hurvitz Brain Sciences Program, Sunnybrook Research Institute, Toronto, ON, Canada; ^5^ Division of Neurology, Sunnybrook Health Sciences Centre and UofT, Toronto, ON, Canada; ^6^ Division of Neurology, Department of Medicine, University of Toronto, Toronto, ON, Canada

## Abstract

**Background:**

In neurodegenerative diseases causing dementia, hippocampal atrophy can lead to cognitive decline and emotional dysregulation. Sleep is crucial for memory consolidation and emotional regulation, with the hippocampus being fundamental in forming episodic emotional memories. The Neuropsychiatric Inventory (NPI) evaluates behavioural symptoms like apathy and aberrant behaviors, while the Pittsburgh Sleep Quality Index (PSQI) assesses sleep quality. This study examined correlations between hippocampal volumes with NPI and PSQI scores in patients with neurodegenerative disease.

**Method:**

Study participant data was obtained retrospectively from the Sunnybrook Dementia Study (SDS) and the Ontario Neurodegenerative Disease Research Initiative (ONDRI). A standardized neuroimaging pipeline was used to measure hippocampal atrophy using HippMapp3r, whole brain atrophy was estimated using the brain parenchymal fraction (BPF), and cerebral small vessel disease was derived from white matter hyperintensity volumes (WMH). NPI and PSQI scores were collected during patient visits. Linear regression and correlation analyses examined associations between hippocampal volumes with NPI and PSQI while controlling for age, sex, education, BPF, and WMH.

**Result:**

In the SDS cohort, partial correlations (*N* = 297) revealed a negative relationship between hippocampal volumes and NPI‐Aberrant scores (ρ=‐0.13, *p* = 0.029). Regression models (*N* = 1027) showed hippocampal volumes were negatively associated with NPI‐Apathy scores (β=‐0.085, *p* = 0.007). In the ONDRI cohort, hippocampal volumes correlated with poorer perceived sleep quality in both the Alzheimer's disease/mild cognitive impairment (ADMCI: N*=*215, ρ=0.26, *p* <0.0001) and cerebrovascular disease (CVD: N*=*331, ρ=0.17, *p* = 0.002) groups. Smaller hippocampal volumes were correlated with longer sleep duration in the Alzheimer's disease/mild cognitive impairment (ADMCI) group (N*=*211, ρ=‐0.33, *p* <0.0001). There was no significant correlation between hippocampal volume and NPI‐apathy scores in ONDRI. Aberrant behaviour was not assessed in ONDRI's NPI‐Q, and PSQI measures were not available in SDS.

**Conclusion:**

Hippocampal atrophy in neurodegenerative diseases may be related to increased apathy, aberrant behaviours, and altered sleep patterns. These findings suggest that diminished hippocampal volumes may impair emotional regulation and introduce compensatory sleep quality mechanisms. Future studies on neurodegenerative diseases with neuroimaging should include full measures of apathy and aberrant behaviour, as well as more objective sleep quality measures (polysomnography or wrist actigraphy) to further explore important relationships with brain atrophy.

